# Early Cambrian renewal of the geodynamo and the origin of inner core structure

**DOI:** 10.1038/s41467-022-31677-7

**Published:** 2022-07-19

**Authors:** Tinghong Zhou, John A. Tarduno, Francis Nimmo, Rory D. Cottrell, Richard K. Bono, Mauricio Ibanez-Mejia, Wentao Huang, Matt Hamilton, Kenneth Kodama, Aleksey V. Smirnov, Ben Crummins, Frank Padgett

**Affiliations:** 1 Department Earth and Environmental Sciences, University of Rochester, Rochester, NY 14627 USA; 2 Department Physics and Astronomy, University of Rochester, Rochester, NY 14627 USA; 3 Laboratory for Laser Energetics, University of Rochester, Rochester, NY 14623 USA; 4 Department Earth and Planetary Sciences, University of California, Santa Cruz, CA 95064 USA; 5 Department Earth, Ocean and Atmospheric Science, Florida State University, Tallahassee, FL 32306 USA; 6 Geomagnetism Laboratory, University of Liverpool, Liverpool, L69 3GP UK; 7 Department Geosciences, University of Arizona, Tucson, AZ 85721 USA; 8 Institute of Tibetan Plateau Research, Chinese Academy of Sciences, Beijing, China; 9 School of Geosciences, University of Oklahoma, Norman, OK USA; 10 Department Earth and Environmental Sciences, Lehigh University, Bethlehem, PA 18015 USA; 11 Department Geol. Mining Engineering and Sciences, Michigan Technological Univ., Houghton, MI 49931 USA; 12 Department Physics, Michigan Technological Univ., Houghton, MI 49931 USA; 13 Present Address: ERM, 1 Beacon St, Boston, MA 02108 USA; 14 Present Address: BAE Systems, 9300 Wellington Rd, Manassas, VA 20110 USA

**Keywords:** Palaeomagnetism, Core processes, Geomagnetism, Geodynamics

## Abstract

Paleomagnetism can elucidate the origin of inner core structure by establishing when crystallization started. The salient signal is an ultralow field strength, associated with waning thermal energy to power the geodynamo from core-mantle heat flux, followed by a sharp intensity increase as new thermal and compositional sources of buoyancy become available once inner core nucleation (ICN) commences. Ultralow fields have been reported from Ediacaran (~565 Ma) rocks, but the transition to stronger strengths has been unclear. Herein, we present single crystal paleointensity results from early Cambrian (~532 Ma) anorthosites of Oklahoma. These yield a time-averaged dipole moment 5 times greater than that of the Ediacaran Period. This rapid renewal of the field, together with data defining ultralow strengths, constrains ICN to ~550 Ma. Thermal modeling using this onset age suggests the inner core had grown to 50% of its current radius, where seismic anisotropy changes, by ~450 Ma. We propose the seismic anisotropy of the outermost inner core reflects development of a global spherical harmonic degree-2 deep mantle structure at this time that has persisted to the present day. The imprint of an older degree-1 pattern is preserved in the innermost inner core.

## Abstract (web summary)

New single crystal paleointensity data show that the geomagnetic field was renewed in the early Cambrian after near collapse in the Ediacaran Period. This implies that the innermost/outermost structure of the inner core formed 450 million yrs. ago.

## Introduction

The growth of the inner core depends on the rate and nature of heat loss to the mantle. The present-day outermost inner core displays hemispherical differences in seismic wave velocity and anisotropy^1–4^. These signatures are thought to reflect variations in the rate of freezing at the outer-inner core boundary that are in turn linked to the degree-2 pattern of core-mantle boundary heterogeneity^5^. A change in seismic anisotropy with depth highlights the possibility that a different, older pattern of core-mantle heat loss is preserved in the structure of the inner core.

A prerequisite for exploring when any change in inner core growth occurred is knowing the nucleation onset age. The geomagnetic field is predicted to be near the weak field state, where core kinetic energy approaches magnetic energy, at the onset of inner core nucleation^6^. Paleointensity measurements consistent with this near weak field state, namely ultralow field strengths, were independently discovered by Bono et al.^7^ in anorthosites of the 565 Ma Sept Îles Layered Mafic Sequence (Quebec, Canada), and subsequently supported by numerous other studies of Ediacaran lavas and dikes^8–10^.

A second, key prediction of inner core growth onset is the rapid recovery of field strength, as latent heat of crystallization and compositional buoyancy renew the geodynamo. The absence of time-averaged paleomagnetic dipole moments of the latest Ediacaran to Early Cambrian age, those data able to provide a synoptic view of the geodynamo^7,11^ (Supplementary Information), has prevented the search for such an increase in field intensity. Thus, a more exact age of nucleation onset, needed to assess inner core growth history, has been wanting.

We sampled well-preserved anorthosites of the early Cambrian Glen Mountains Layered Complex (GMLC)^12^ of the Wichita Mountains (Oklahoma) to fill this data gap^7^ (Methods, Supplementary Fig. 1). U-Pb zircon geochronology yields an age of 532.49 ± 0.12 Ma for these anorthosites^13^. Fe-Ti oxide needles have been reported in plagioclase from GMLC anorthosites^14^ similar to other occurrences yielding high fidelity paleomagnetic and paleointensity data^7,15^, suggesting that these rocks are ideal candidates for application of the single-crystal paleointensity (SCP) technique^7,11,16–22^ (Methods). Specifically, in this application single silicate crystals can better isolate single-domain (SD) magnetic grains, which are the most robust recorders of the geomagnetic field.

## Results

### Rock magnetism

All rock magnetic, paleomagnetic, and paleointensity measurements were performed in the Paleomagnetic Laboratories at the University of Rochester (Methods). We start with an analysis of bulk rock anorthosite samples. Magnetic susceptibility versus temperature data (*K*-T curves, −190 to 700 °C) measured in air and argon demonstrate that the dominant magnetic carriers are magnetite and titanomagnetite (Supplementary Fig. 2). Magnetic hysteresis and first-order reversal curves (FORCs) provide evidence for mixtures of a single domain (SD), pseudosingle domain (PSD), and multidomain (MD) grains (Supplementary Fig. 3, Supplementary Information). Stepwise alternating field (AF) demagnetization yields well-defined characteristic remanences (ChRMs) from two sites (Supplementary Fig. 4a–d and Supplementary Table 1), whereas one site was excluded from further analysis due to inconsistent directions (Supplementary Information).

The two selected sites have different polarities; an evaluation of randomness^23^ applied to these data yields a result (*V*_*w*_ = 1.9) smaller than the critical value (*V*_*c*_ = 7.9) at the 95% confidence level. This indicates that the null hypothesis of a common mean direction between sites cannot be rejected, representing a positive reversal test (Methods, Supplementary Fig. 4e). Samples from these sites yield a mean paleomagnetic direction of D = 236.2^o^, I = 7.3^o^, N = 12, *k* = 51, and *α*_95_ = 6.1^o^, where D is declination, I is inclination, *k* is the best estimate of the precision parameter *κ*, and *α*_95_ is the radius of 95% confidence. This direction agrees with the results of a prior paleomagnetic study^24^ (Supplementary Fig. 4f–g), and together with the reversal test results indicates that the anorthosites we have sampled carry a primary magnetic remanence.

**Fig. 1:**
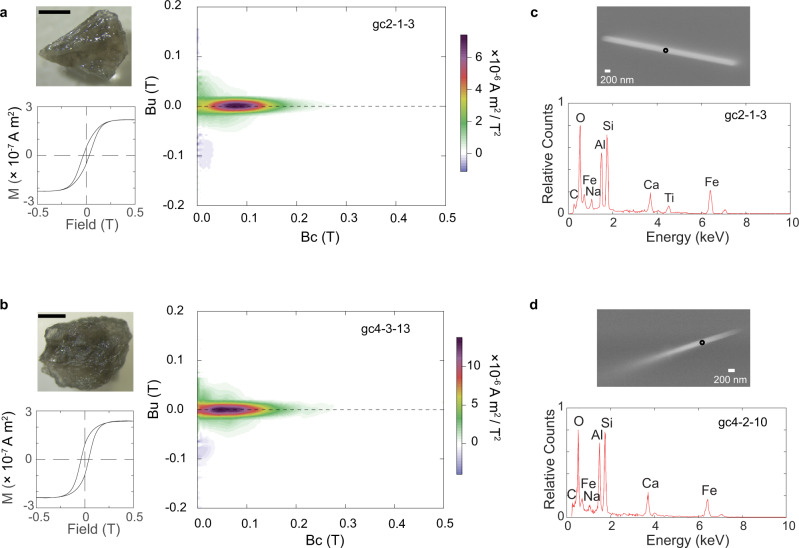
Rock magnetism and SEM analysis of plagioclase crystals from the GMLC anorthosites. **a**, **b** Images of the measured plagioclase crystal (upper left, scale bar 1 mm), magnetic hysteresis loop (bottom left), and first-order reversal curves (FORC) diagrams (right). FORC diagrams use the following smooth factors: **a** Sc0 = Sb0 = 4, Sc1 = Sb1 = 7, *λ*_*x*_ = *λ*_*y*_ = 0.1; **b** Sc0 = Sb0 = 4, Sc1 = Sb1 = 10, *λ*_*x*_ = *λ*_*y*_ = 0.1. **c**, **d** Backscatter scanning electron microscope images of elongated magnetic inclusions observed in the plagioclase crystals (upper) and their corresponding energy-dispersive X-ray spectroscopy (EDS) spectra (bottom) in 20 keV. EDS spots analyzed are marked as black circles in the images.

Having identified suitable sites, we next investigate the magnetic properties of plagioclase from the anorthosite. Plagioclase crystals 1 to 4 mm in size were separated from bulk rock samples for rock magnetic, electron microscope, and paleointensity analyses. Magnetic hysteresis curves and FORC plots show that these crystals have non-interacting SD or PSD behavior without the relatively large MD component seen in some bulk rock samples (Site 2: *M*_*r*_/*M*_*s*_ = 0.26 ± 0.06, *B*_*cr*_/*B*_*c*_ = 2.68 ± 0.37; Site 4: *M*_*r*_/*M*_*s*_ = 0.42 ± 0.07, *B*_*cr*_/*B*_*c*_ = 1.75 ± 0.26; Fig. 1a, b and Supplementary Fig. 5a, b). These data confirm the SCP approach and selection methods. Light and scanning electron microscope (SEM) analyses, the latter employing energy-dispersive spectroscopy (EDS) (Fig. 1c, d), reveal the presence of needle-shaped magnetite and titanomagnetite inclusions. These occur along multiple crystallographic axes (Supplementary Fig. 5c, d). The needles are typically ~200 nm in width and several micrometers long; some are finer, 50–100 nm wide (Supplementary Fig. 5e, f). They appear to be compositionally homogeneous, without evidence for magnetite-ulvöspinel unmixing, suggesting that they can carry a primary thermal remanent magnetization (TRM)^25^. Isolated, much larger magnetite/titanomagnetite inclusions several microns in size are occasionally observed in the GMLC anorthosite plagioclase, highlighting the need to screen crystals bearing visible inclusions from paleointensity analysis^19,26^ (Methods). The SEM observations are important in defining the typical crystallographic occurrence of the magnetic grains, but they sample only a small volume. However, the magnetic hysteresis data probe the entire crystal measured. Thus, magnetic hysteresis curves and FORC plots, together with the SEM data, provide comprehensive documentation of the dominance of robust magnetic mineral recorders in the anorthosite plagioclase.

### Paleointensity

Having documented the dominance of magnetic particles meeting Thelliers’ criteria for ideal magnetic recording^27,28^, we now apply SCP techniques^19,20^ including CO_2_ laser heating^29^. We start with total TRM (TTRM) experiments^30^ (Methods) to provide unblocking temperature references for later study. The highest unblocking temperature observed in these experiments ranges from 400 to 520 °C, whereas data from 3 crystals, measured using an applied field of 30 μT, provide an approximate paleointensity of 13.9 ± 7.1 μT (Supplementary Fig. 6 and Supplementary Table 2).

**Fig. 2:**
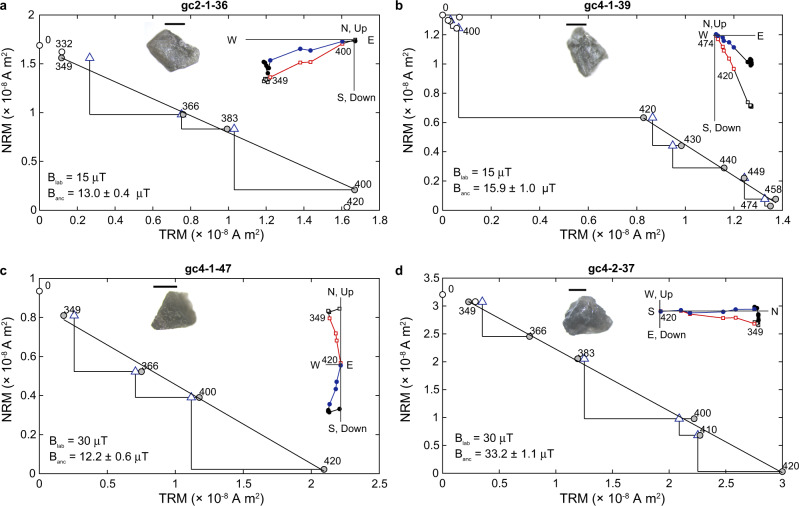
Thellier–Coe paleointensity experiments of single plagioclase crystals from the Glen Mountains Layered Mafic Complex anorthosites. **a**–**d** Natural remanent magnetization (NRM) lost versus thermal remanent magnetization (TRM) gained (circles) and partial thermal remanent magnetization checks (triangles). All labeled points are ^*o*^C. Gray circles are steps used in fit (applied field B_lab_ and paleointensity value B_anc_ shown in lower left). Insets: crystal measured shown in the top center with 1 mm scale bar. An orthogonal vector plot of field-off steps is shown in the upper right. Squares are the vertical projection of the magnetization; circles are the horizontal projection. Temperature ranges used in the paleointensity fit are labeled and symbols are in color.

We next performed Thellier–Coe experiments with partial TRM (pTRM) checks^28,31^ on 117 single plagioclase crystals to fully investigate the paleointensity signal (Methods). These results were assessed by examining the natural remanent magnetization (NRM) lost versus TRM gained (Fig. 2 and Supplementary Fig. 7). Selection criteria follow those of ref. ^32^ and ref. ^7^ (Supplementary Table 3) and are summarized as follows: (i) at least four steps on the Arai plot must be included in fitting the paleointensity slope; (ii) the correlation coefficient (*R*^2^) of the major axis fit should be greater than 0.9; (iii) the field-off characteristic remanent magnetization (ChRM) vector must trend to the origin of orthogonal vector plots (equivalent to DANG of Tauxe (see ref. ^7^) <15^o^); (iv) the ChRM vector must have a small maximum angular deviation (MAD) <15^o^; and (v) the pTRM check should be within 10% of the reference value (the equivalent of DRAT^33^ ≤10%).

Seventeen samples are accepted as reliable paleointensity estimates (Supplementary Information, Fig. 2, Supplementary Fig. 7, and Supplementary Table 3). Of these, 13 passes all criteria and are ranked as an “A” category result. Three samples do not meet one criterion (one with low *N* = 3, one with a pTRM check passing only at 17.7%, and one with *R*^2^ = 0.879) and are assigned to category “B”. One result did not pass two criteria (low *N* = 3 and a DRAT = 10.3%) and is considered a category “C” result (see Supplementary Information and Supplementary Table 3).

We applied both 15 and 30 μT laboratory fields in our Thellier–Coe experiments to check for nonlinear field acquisition^34^. The averaged paleointensities for the two applied fields (17.7 ± 4.4 μT, *n* = 8, applied field of 15 μT; 19.9 ± 6.2 μT, *n* = 9, applied field of 30 μT) are indistinguishable within one standard deviation (Supplementary Table 3). Overall accepted results from Site 2 (17.5 ± 4.7 μT, *n* = 7) and those of Site 4 (19.8 ± 5.9 μT, *n* = 10) are also indistinguishable within one standard deviation. For all accepted measurements of the two sites (*n* = 17), the average paleointensity is 18.9 ± 5.4 μT, which matches the expectation of TTRM results.

**Fig. 3:**
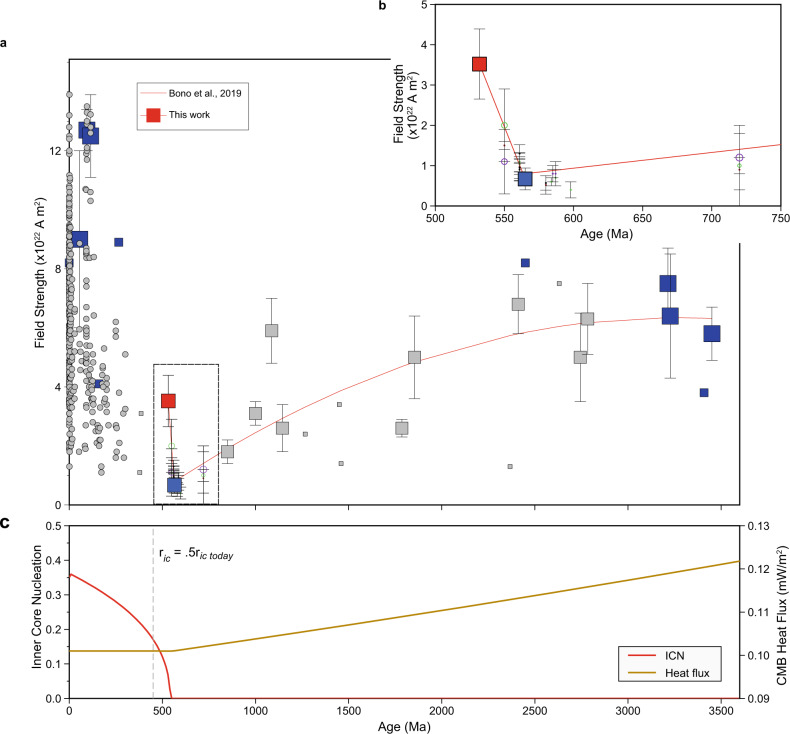
Early Cambrian field renewal and inner core growth. **a** Field strength constrained from select Thellier (thermal) single-crystal paleointensity (SCP) studies (blue squares) and bulk rock studies (gray squares) updated from ref. ^7^, with new early Cambrian SCP result (red square) reported here. Large squares are time-averaged paleomagnetic dipole moments; small squares are virtual dipole moments (VDMs). Gray circles are select Phanerozoic VDMs from ref. ^7^. Field evolution model (3450 Ma to 565 Ma, red line) is weighted second-order polynomial regression of Precambrian field strength data from ref. ^7^; 565 to 532 Ma trend connects the Ediacaran paleomagnetic dipole moment of ref. ^7^, and the new Early Cambrian paleomagnetic dipole moment from this work. Error bars are 1*σ*. **b** Ediacaran to Cambrian field strength evolution corresponding to a dashed rectangle in **a**. Open circles are results from non-Thellier methods (non-thermal and thermal) and their sizes are weighted by the number of cooling units. Key: green, microwave method; purple, Shaw method; black, Wilson method. Brown open circles are Thellier thermal results. **c** Thermal model^49^ showing inner core nucleation (ICN) age versus core-mantle boundary (CMB) heat flux. Dashed vertical line is the time when the inner core (radius = *r*_ic_) is 50% of its current size (*r*_ic__modern_), the location of the change observed in seismic anisotropy^1^.

We conducted anisotropy tests (Methods) which yielded anisotropy factors^35^ varying from 0.7 to 1.4. We also considered the effect of cooling rate, using a cooling time constrained by GMLC geochronology of ~500 kyr^13^. This cooling time indicates that the GMLC anorthosites record the time-averaged paleomagnetic field, but that our raw data may overestimate the intensity^36–38^ by a factor of ~1.5. After both cooling rate and anisotropy corrections, the final paleointensity is 13.7 ± 3.4 μT. Using a paleolatitude of 3.7^∘^N derived from our AF demagnetization results (reference site at 34.82^o^N, 98.95^o^W), the corresponding paleomagnetic dipole moment (PDM) is 3.5 ± 0.9 × 10^22^ A m^2^ (Fig. 3a).

## Discussion

The PDM from the GMLC anorthosite plagioclase is 5 times higher than that of the ultralow Ediacaran field at 565 Ma^7^, and indicates the field had recovered to this field strength in ≤33 Myr (Fig. 3a, b). Available instantaneous records of the latest Ediacaran field strength^39^ are consistent with this value (Supplementary Information and Supplementary Table 4). This relatively rapid recovery agrees with expectations of new energy sources to power the geodynamo from inner core growth. We note that the rapid increase is predicted by thermal evolution/geodynamo models employing scaling laws^6,40,41^.

We note that the time-averaged nature of the 565 and 532 Ma data separate the observed behavior from fluctuations of the field seen in some numerical simulations of the field that occur on short times scales (i.e., a small fraction of a magnetic diffusion time)^42^. A caveat on any interpretations of the ancient intensity of the geodynamo is the sparseness of the robust dataset, especially that defined by time-averaged data and magnetic carriers with documented non-interacting single-domain magnetic mineral carriers. Notwithstanding this limitation, we note that this distinctive pattern of change from the near weak field state to stronger fields defined by our new data is not seen in the Mesoproterozoic paleointensity record^43,44^, previously proposed as a time of inner core growth^45^, and its identification here now more firmly establishes the latest Ediacaran/earliest Cambrian as the time of growth onset. The onset age of 550 Ma, midway between the available Ediacaran and earliest Cambrian PDMs, implies a high core thermal conductivity (89–121 W mK^−1^)^7^. If there is delayed nucleation of the inner core^46^, the paleointensity history suggests it was limited to the ultralow geomagnetic field strength, which may have spanned several tens of millions of years during the Ediacaran Period (Fig. 3b).

Although model details differ^2–4,47,48^, recent analyses show that the slow direction of seismic anisotropy changes from an equatorial orientation to 54^o^ relative to the rotation axis, at a radius of ~650 km^1^. A thermal evolution model^49^ using a ~550 Ma onset age suggests the inner core would have grown to this size by 450 ${}_{-15}^{+10}$ Ma (Fig. 3c, Methods). Because the present-day degree-2 deep mantle structure sets a boundary condition on current inner core growth^5^, we assign ~450 Ma as the time when this deep mantle structure formed, leading to the outermost inner core seismic signature.

Reconstructions of deep mantle structure based on plate motions are limited by the loss of oceanic crust >200 million years old to subduction, but the inner core structure can provide a constraint on this history. In particular, prior ideas on when the deep mantle degree-2 pattern originated vary from ≥540 to <330 Ma and stem from the debate on whether the Atlantic and Pacific large low shear velocity provinces (LLSVPs) formed from post-Pangea subduction^50^ or are much older^51^. Our analyses, together with inferences from recent plate reconstructions^52^, suggest that the present-day degree-2 structure formed before the assembly of Pangea, but that the African LLSVP is no older than ~450 Ma. The seismic anisotropy of the innermost inner core likely records dominance of subduction in the paleo-Pacific hemisphere (degree-1)^52^ prior to 450 Ma.

Multiple phases of iron may exist in the inner core^53,54^, but there is no compelling reason from iron phase diagram data for a major change at a radius of 650 km. A recent posit that metastable body-centered (bcc) iron may be a precursor to stable hexagonal close-packed iron, thought to be the dominant phase in the inner core, can help address questions about initial crystallization^54^. But melting curves indicate that the bcc phase could persist only at conditions near the very center of Earth^54^ rather than at the change in seismic anisotropy^1^.

The efficiency of the magnetic field since 450 Ma has been governed by zonal and non-zonal variations in core-mantle boundary heat flow^55^ superimposed on the dominant degree-2 deep mantle structure, as evidenced by variations in paleointensity and reversal frequency since the early developments of Pangea in the Devonian on ~200 Myr time scales^7,11,17,19,20,56–58^. But post ~450 Ma convection has not changed the dominant degree-2 mantle structure, which remains as the principal factor controlling the nature of inner core growth.

## Methods

### Sample collection and preparation

We collected large (≥30 cm^3^) GMLC anorthosite blocks that were oriented by magnetic and Sun compasses from each of three sites Site 2 (34.79^o^N, 98.86^o^W), Site 3 (34.86^o^N, 99.01^o^W), and Site 4 (34.82^o^N, 98.95^o^W). Additional large unoriented samples were collected adjacent to the oriented samples to ensure sufficient material far from the influence of weathering was available for study. Outcrops were scanned with a Brunton compass to test for the presence of lightning strikes (none were observed at these sample locations). Samples for *K*-T measurements were obtained by crushing subsamples into a powder using non-magnetic tools, whereas those for magnetic hysteresis were mm-sized chips. For directional studies, 2.54 cm cores were drilled from oriented samples at the University of Rochester. To prepare the plagioclase crystal samples for magnetic hysteresis, microscopy, and paleointensity studies, we crushed the anorthosites with non-magnetic tools, and then used a Nikon SMZ800 light microscope to pick clean plagioclase crystals 1–4 mm in size, avoiding those crystals with visible opaques under low power (10x) magnification. The naming convention for single-crystal specimens is as follows: gc-a-b-c′, a is the site, b is the hand sample, and c′ is the crystal. For groundmass, the convention is gc-gm-e-f-g′, where e is the site, f is the hand sample, and g′ is the groundmass specimen. For paleomagnetic analysis and magnetic susceptibility measurements of whole rocks, the convention is gc-w-x-yz where w is the site, x is the hand sample, y is the drilled core, and z is the specimen cut from the core.

### Rock magnetism and paleomagnetism

Magnetic volume susceptibility versus temperature (*K*-T) data on bulk anorthosite subsamples (~0.6–1cc) were collected using a KLY-4S Agico Kappabridge. Magnetic hysteresis data and FORCs for both plagioclase crystals and bulk specimens were measured using a Princeton Measurements Corporation Alternating Gradient Force Magnetometer (AGFM). The maximum applied field used for the individual magnetic hysteresis curve was 500 mT. FORC data were collected on single crystals as follows: Each FORC measurement is composed of 100 FORCs; the saturation field was 1 T, with a field increment of 10 or 12 mT, and an averaging time of 250 ms was used; *B*_*c*_ ranges from 0 to 0.5 T and *B*_*u*_ from −0.5 to 0.1 T. FORC data collected on groundmass use the following parameters: Each FORC measurement composed of 100 FORCs, the saturation field used was 1 T, with a field increment of 12 mT and an averaging time of 500 ms; *B*_*c*_ ranges from 0 to 0.5 T and *B*_*u*_ from −0.3 to 0.3 T. FORC data were processed using FORCinel^59^ and the VARIFORC smoothing algorithm^60^. A Magnon 300 AF demagnetizer was used for demagnetizations of oriented whole rocks. Increments were ≤5 to 20 mT, and then 10–20 mT to the maximum applied demagnetization fields (100–190 mT). Magnetic remanence measurements (for AF demagnetization, and paleointensity analyses described below) were made using the 2G three-component DC SQUID magnetometer with high resolution sensing coils in the magnetically shielded room (ambient field <200 nT) in the Paleomagnetism Laboratory at the University of Rochester. Demagnetization and reversal test data were processed using the PmagPy GUI software of ref. ^61^; the latter are useful for further demonstrating a primary magnetic remanence expected for the single domain recorders present^7,28^.

### Paleointensity measurements and analyses

Single plagioclase crystals were mounted on 5-mm quartz rods using sodium silicate. These sample preparation materials have negligible magnetizations as demonstrated by internal tests in the University of Rochester lab and in interlab analyses^62^. For all thermal treatments, single crystals were heated for 90 s using either a Synrad v20 or v40 CO_2_ laser (both in the magnetically shielded room at the University of Rochester). The 10.6 μm wavelength couples extremely well with silicates; temperature calibrations follow prior studies and employ infrared pryometers^29,63^. For TTRM experiments, plagioclase crystals were stepwise thermally demagnetized until ~90% of the natural remanent magnetization (NRM) was removed. Thereafter, a total TRM was applied using a 30 μT applied field along the laboratory + Z axis. This TTRM was then removed by stepwise thermal demagnetization. The NRM and TTRM demagnetization curves are compared to derive a paleointensity estimate^30^. For Thellier–Coe experiments, samples were heated in a field-off environment to ~300 °C (an unblocking temperature range where overprints are removed, as seen in the TTRM experiments), with ~100 °C steps, after which smaller temperature steps (~10–30 °C) were used. In general, there is a desire to minimize the number of temperature steps because, even with the short heating durations achievable with CO_2_ laser heating, each additional step increases the chances of alteration. A 15 or 30 μT field was applied along the laboratory +Z axis in field-on steps. Initial processing of the paleointensity data utilized Pmag GUI software^61^, whereas final values were calculated by analyzing each dataset individually. Although not used in our selection criterion we report FRAC values, the ratio of vector difference sums of NRM defining the best fit line, and the NRM of all steps measured:

1 $${{{{{\mathrm{FRAC}}}}}}=\frac{\mathop{\sum }\nolimits_{i = {{{{{\mathrm{start}}}}}}}^{{{{{{\mathrm{end}}}}}}-1}\left|{{{{{\mathrm{NR}}}}}}{{{{{{\mathrm{M}}}}}}}_{i+1}-{{{{{\mathrm{NR}}}}}}{{{{{{\mathrm{M}}}}}}}_{i}\right|}{\left|{{{{{\mathrm{NR}}}}}}{{{{{{\mathrm{M}}}}}}}_{{n}_{{{{{{\mathrm{max}}}}}}}}\right|+\mathop{\sum }\nolimits_{i = 1}^{{n}_{{{{{{\mathrm{max}}}}}}}-1}\left|{{{{{\mathrm{NR}}}}}}{{{{{{\mathrm{M}}}}}}}_{i+1}-{{{{{\mathrm{NR}}}}}}{{{{{{\mathrm{M}}}}}}}_{i}\right|}$$

where $\left|{{{{{\mathrm{NR}}}}}}{{{{{{\mathrm{M}}}}}}}_{i}\right|$ denotes the length of the NRM vector at the *i*th step, *n*_max_ is the last step measured, and start and end represent the starting and ending temperature steps of the best line fit.

We used the method of Veitch^35^ to assess magnetic remanence anisotropy of the plagioclase crystals. Each assessment was conducted at a temperature step used for constraining paleointensity for that crystal. Partial TRMs were imparted in the +Z, −Z, +Y, −Y, +X, and −X directions, and used to calculate a TRM susceptibility tensor and anisotropy correction factor^35^ *f*. We also require that these data pass two internal consistency tests. The factor *f* calculated using pTRMs acquired only in the positive laboratory directions (i.e., +Z, +Y, +X) must be within 20% of that calculated using the pTRMs acquired only in the negative laboratory field directions. In addition, pTRMs of corresponding positive/negative laboratory field direction pairs must be within 30% of each other. We use cooling rate correction formulations^36^ that, in turn, employ an anorthosite cooling time constrained by the high precision U-Pb ages of the GMLC anorthosite and later Roosevelt Gabbro^13^ of ~500 kyr.

### Thermal model

The calculations shown in Fig. 3c follow the methods described by Nimmo^49^. Prior to inner core nucleation, the core-mantle boundary (CMB) heat flux is fixed so as to generate a constant entropy production rate (a measure of the possible dynamo strength) of 5 MW/K. Once inner core nucleation begins, the CMB heat flux is held constant but the rate of entropy production increases because of the latent heat and buoyancy terms. Parameter values are the same as in Model 2 of Nimmo^49^ except that the latent heat of the core was increased by 20% to produce an inner core age (i.e., 550 Ma) consistent with the observations. To derive bounds on the age when the inner core radius reached 1/2 its current size, we use two inner core growth onset times close to the ages of the paleomagnetic dipole moments used to define the increase in field strength (530 and 570 Ma, and latent heat increases of 7 and 33%, respectively).

## Supplementary information

Supplementary Information

## Data Availability

Data presented here are available in the Earthref (MagIC) database (earthref.org/MagIC/19525; 10.7288/V4/MAGIC/19525).
