# Supplementary material for: Early Cambrian renewal of the geodynamo and the origin of inner core structure
Source: Nat Commun. 2022 Jul 19;13:4161. doi: 10.1038/s41467-022-31677-7 (PMC9296475; doi:10.1038/s41467-022-31677-7)
Supplement: Supplementary file 1 — Supplementary Information [file 41467_2022_31677_MOESM1_ESM.pdf]

May 31, 2022

Supplementary Information

Early Cambrian renewal of the geodynamo and the origin of inner core structure

Tinghong Zhou, John A. Tarduno, Francis Nimmo, Rory D. Cottrell, Richard K. Bono, Mauricio Ibanez-Mejia, Wentao Huang, Matt Hamilton, Kenneth Kodama, Aleksey V. Smirnov, Ben Crummins, Frank Padgett III

1.0 Time-averaged paleomagnetic field.

The geomagnetic field can be as described by the scalar potential $\psi_m(r,\theta,\phi,t)$:

$$\Psi_m(r,\theta,\phi,t) = \frac{r_e}{\mu_o}\sum_{l=1}^{\infty}\sum_{m=0}^{l}\left(\frac{r_e}{r}\right)^{l+1} P_l^m cos\theta\,[g_l^m(t)cosm\phi + h_l^m(t)sinm\phi] \tag{1}$$

where P_l^m are partially normalized Schmidt functions, l and m are spherical harmonic degree and order, respectively, r_e is the radius of Earth and the Gauss coefficients $g_l^m(t)$ and $h_l^m(t)$ describe the spatially and time-varying fields. There are two salient issues related to time averaging of the Ediacaran to early Cambrian geomagnetic field. First, the field recorded at any location is expected to vary substantially due to secular variation and second, the Ediacaran field is thought to exhibit a hyper-reversal frequency. Accordingly, averaging on time scales greater than many tens-of-thousands of years is needed to exceed the influence of these short term influences and learn about the axial dipole signal (g_1^0).

2.0 GMLC anorthosite site geology.

We sampled to reproduce the results of Roggenthen et al.[1], who performed a comprehensive sampling, but did not fully demagnetize their samples using modern techniques. We sampled 4 localities (Supplementary Figure 1) in the "Western" Wichita's of Roggenthen et al.[1]. Site 1 was examined but not sampled for paleomagnetism. Site 3 is close to the cross-cutting Cold Springs Breccia; directions are scattered confirming the inference in Roggenthen et al.[1] that sites near this contact could be affected by this later felsic magmatism. At Sites 2 and 4 we are able to reproduce the directions isolated by Roggenthen et al.[1], using complete demagnetizations.

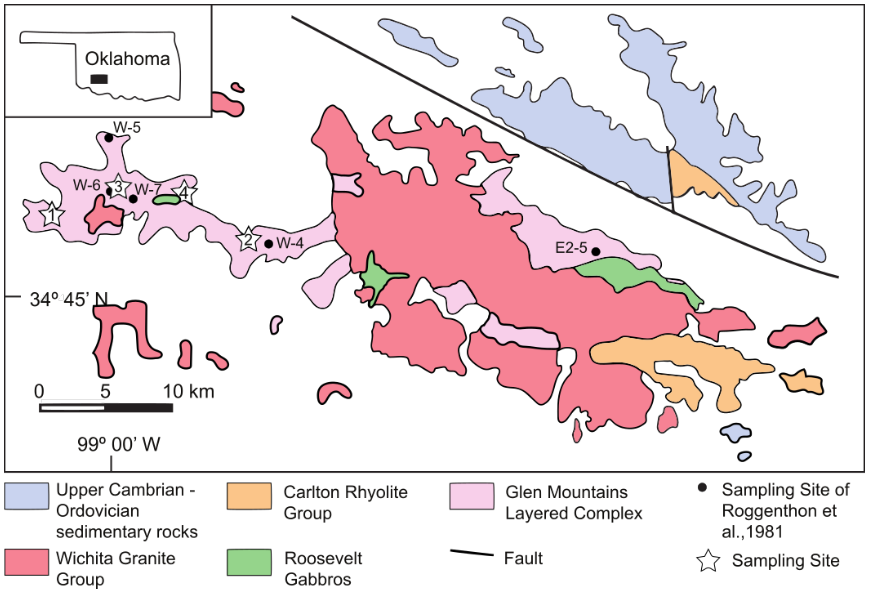

Supplementary Figure 1. Sampling sites of the GMLC anorthosites and summary geologic map after Hanson et al.[2]. Stars: investigation and sampling sites considered here. Black circles: approximate location of sampling sites of Roggenthen et al.[1], estimated from their Fig. 1.

3.0 Rock magnetic, paleomagnetic and paleointensity data.

Our bulk magnetic susceptibility data, collected on crushed ~0.6-1 cc samples, arguably best reflect the compositions of GMLC bulk samples (Supplementary Figure 2). Because the mineralogy of the GMLC anorthosite is dominated by plagioclase, small mm-sized "bulk" samples measured for hysteresis are essentially plagioclase with other minor components (e.g. clinopyroxene and opaques). Accordingly, the magnetic hysteresis parameters of these bulk samples sometimes mirror those of the isolated plagioclase (Supplementary Figure 3). But for other samples they diverge toward the MD state, reflecting varying amounts of these non-feldspar components. Our paleomagnetic analyses confirm the prior results of Roggenthen et al.[1].

One hundred single crystal Thellier experiments failed to yield data passing our selection criteria. Reasons for this failure are as follows: 34 samples lost 90% or more of their natural remanent magnetization at relatively low temperature steps (i.e., below 332 ^{o}C) and continuation of the experiment was impractical; 23 samples lacked sufficient Arai plot data (i.e. $N<4$); 41 samples failed to pass R^2 and/or the DRAT criteria; and 2 experiments failed because the crystal detached from the sample holder during heating. Three of the accepted samples (Category B) fail one of the selection criteria, but pass all others, and the numerical value of the failed criterion is close to passage, or N=3 (see Supplementary Table 3). One accepted case (Category C) has N=3, and fails one other criterion, but with a value very close to passage.

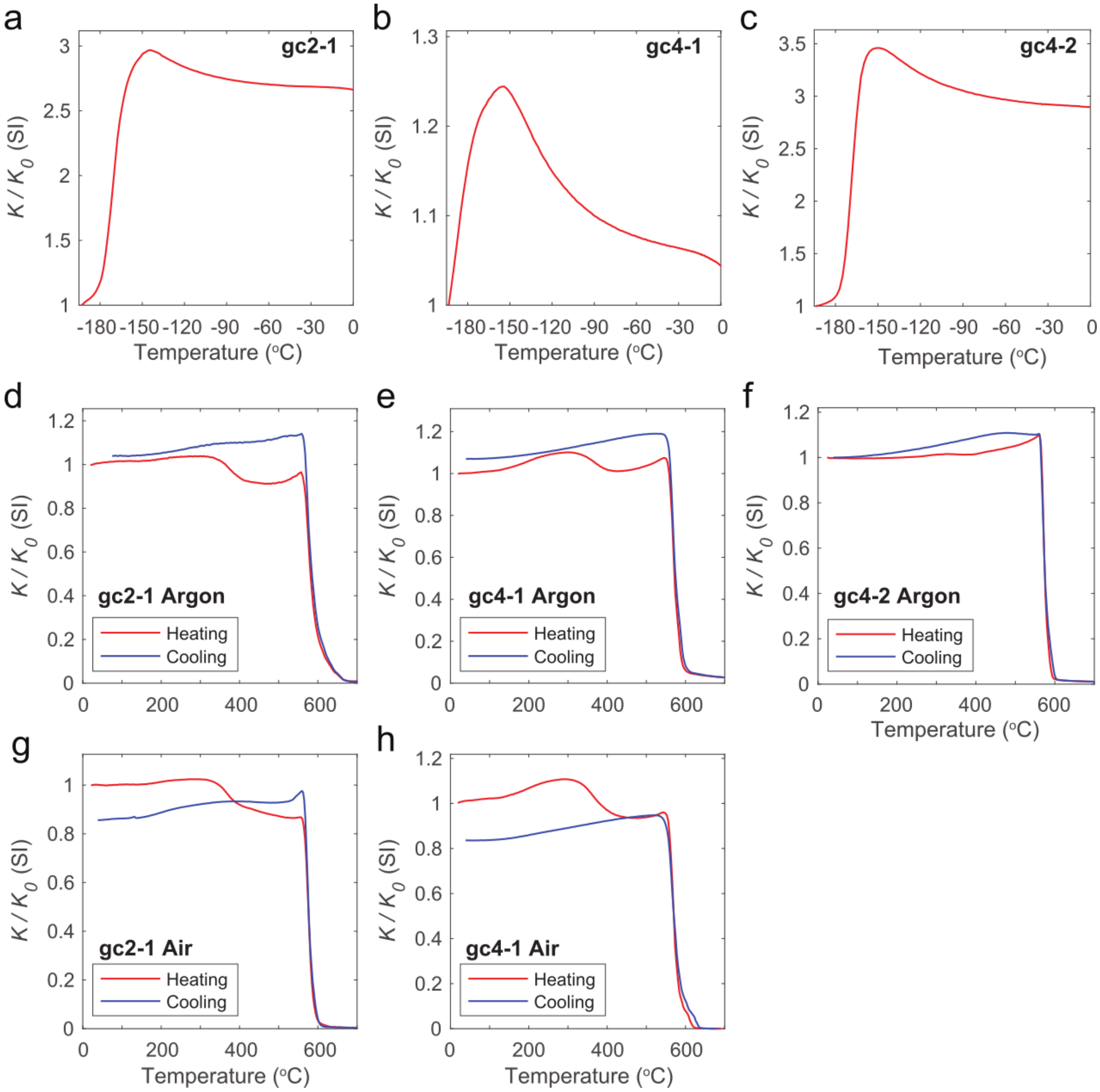

Supplementary Figure 2. GMLC anorthosites magnetic susceptibility data. a-c Low temperature K-T curves from bulk samples of the GMLC anorthosites (Methods). All samples presented here are warmed from low temperature in air. **d-h** High temperature K-T curves from representative bulk rock samples of the GMLC anorthosites. Red, heating curve; blue, cooling curve. The heating atmosphere (in air or Argon) is labeled in each plot.

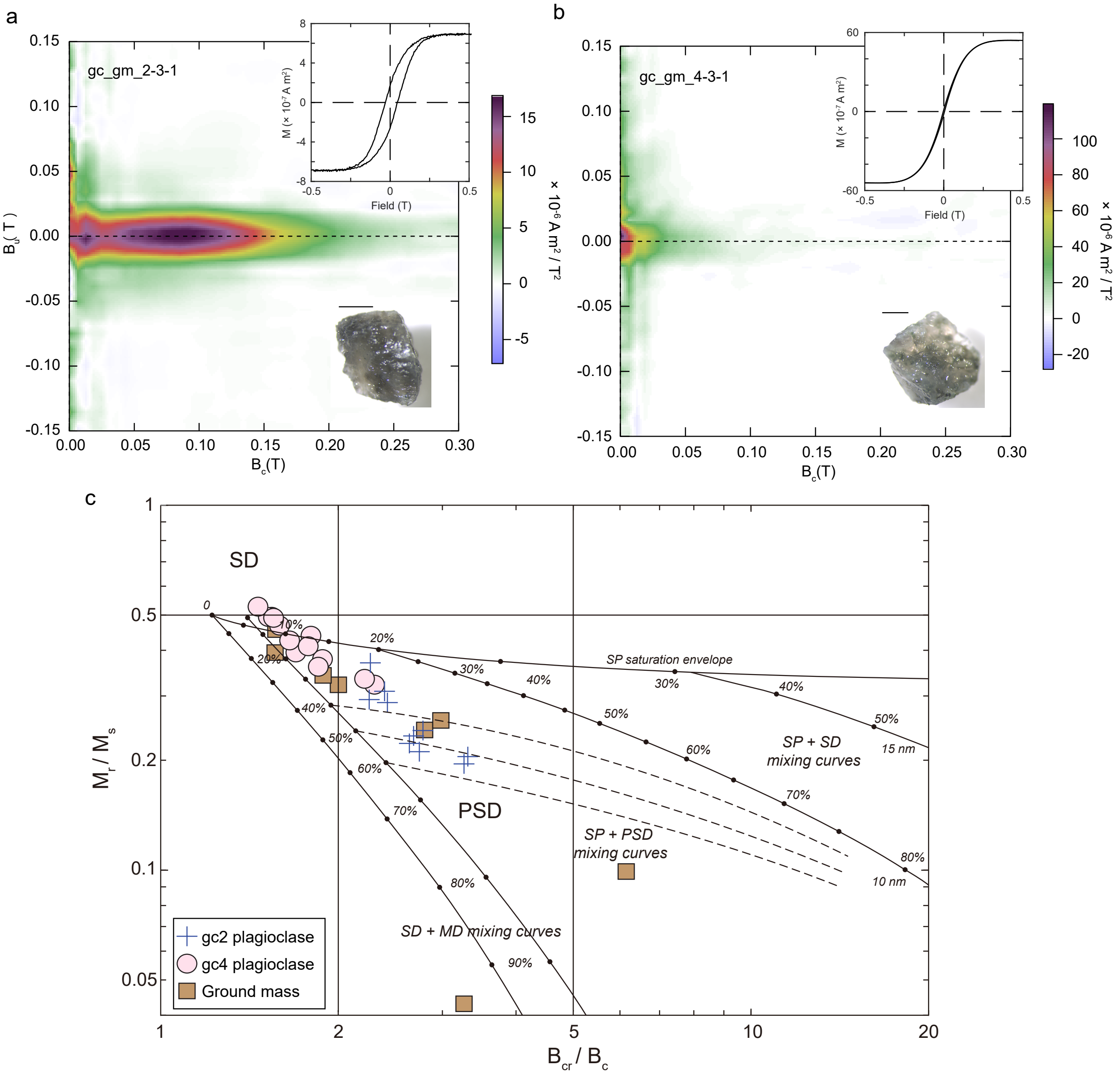

Supplementary Figure 3. Magnetic hysteresis measurements for GMLC anorthosites groundmass specimens and summary magnetic hysteresis parameters for GMLC single plagioclase crystals and groundmass specimens. **a-b** For each groundmass specimen analyzed: Bottom right, image of crystal with 1 mm scale bar; Top right, magnetic hysteresis loop; Left, FORC diagram (see Methods). FORC smoothing parameters are as follows: **a**, Sc0 = Sb0 = 5, Sc1 = Sb1 = 10, λx = λy = 0.1; **b**, Sc0 = Sb0 = 4, Sc1 = Sb1 = 10, λx = λy = 0.1. **c** Day plot of the ratio of saturation remanence (M_r) to saturation magnetization (M_s) versus coercivity of remanence (B_{cr}) to coercivity (B_c), with mixing curves after Dunlop[3].

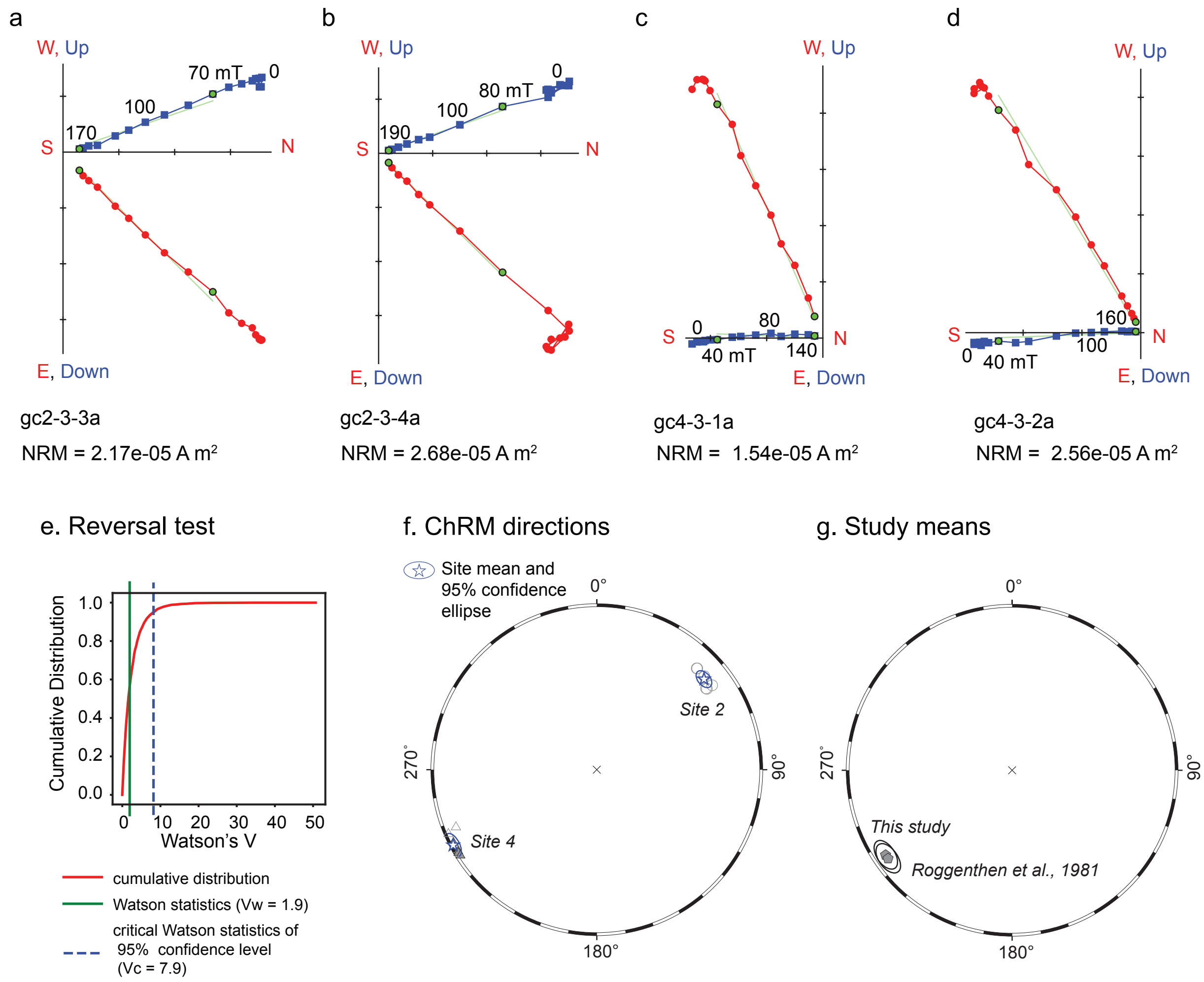

Supplementary Figure 4. Demagnetization results from bulk anorthosite specimens of the GMLC. a-d Orthogonal vector plots of stepwise alternating field demagnetization (in geographic coordinates; field relationships are compatible with negligible stratigraphic corrections for the sites sampled). Red: horizontal projection of the magnetization (declination); blue: vertical projection of the magnetization (inclination). **e** Watson reversal test[4] for the isolated characteristic remanent magnetizations (ChRMs) of Site 2 and Site 4. Red line: cumulative distribution. Solid green line: The Watson statistics (Vw); Vw = 1.9. Dash blue line: critical Watson statistics of 95% confidence level (Vc); Vc = 7.9. **f** ChRMs of the AF demagnetized specimens. **g** Comparison of study means.

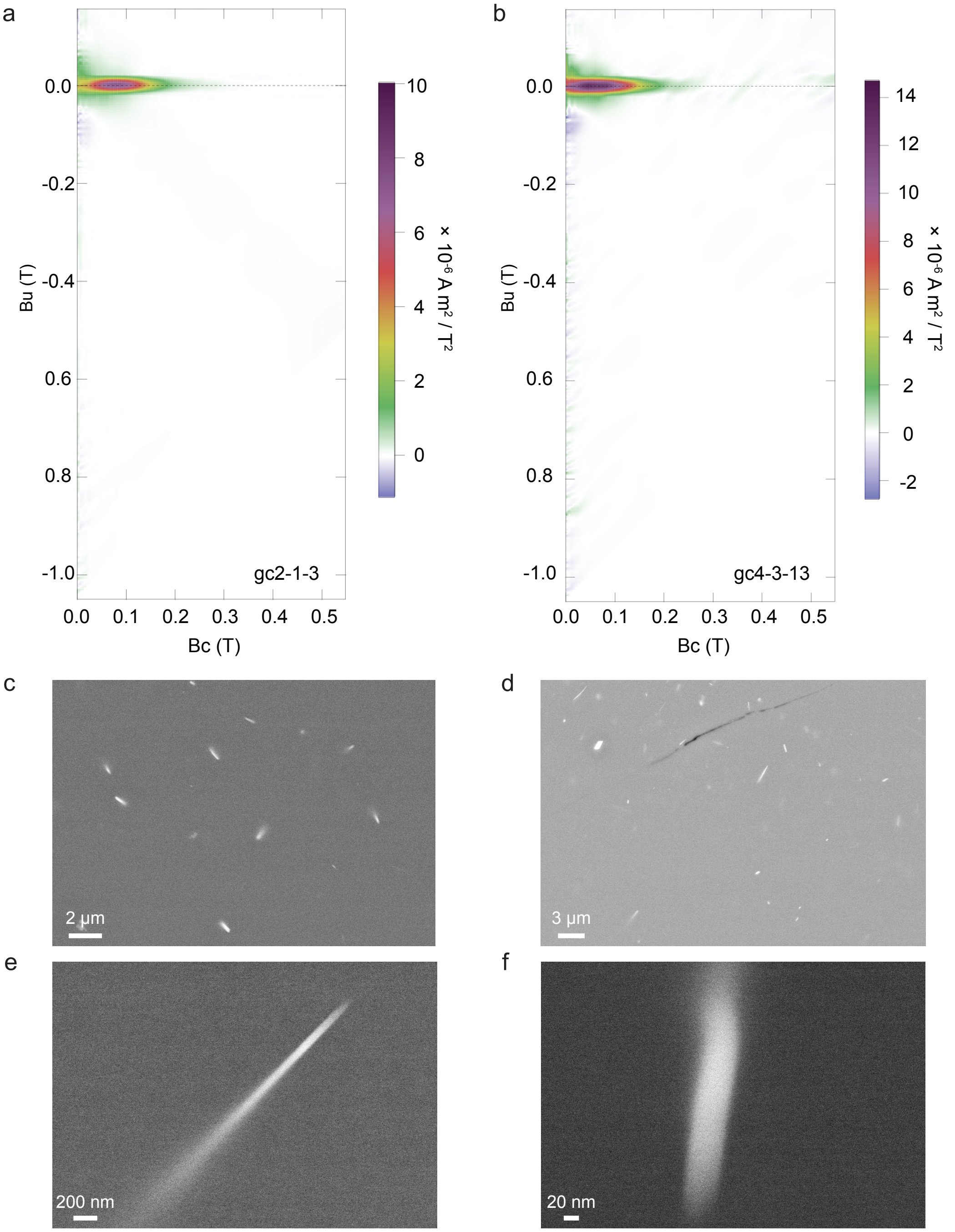

Supplementary Figure 5. Expanded FORC plots and additional SEM images of magnetic needles in plagioclase crystals from the GMLC anorthosites. a-b Expanded FORC plots (see Figure 1 of the main text for smoothing parameters). **c-f** SEM backscatter images with acceleration voltage of 20 keV. Plagioclase crystals are as follows: **c**, gc2-1-4; **d**, gc4-2-9; **e**, gc4-1-11; **f**, gc4-1-11.

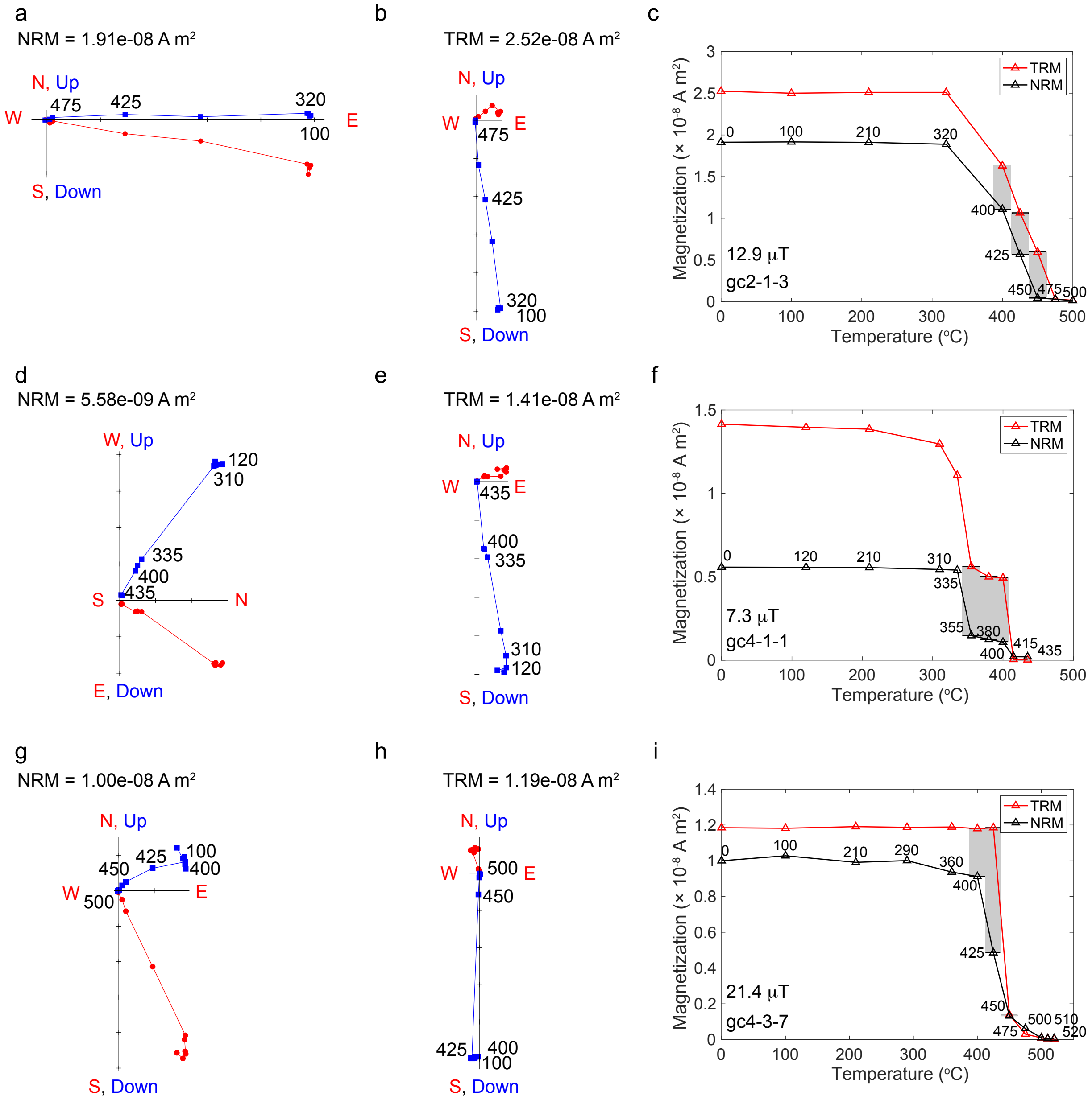

Supplementary Figure 6. TTRM experiment on plagioclase crystals from the GMLC anorthosites. Left: orthogonal vector plots of NRM thermal demagnetization (in specimen coordinates); Middle: orthogonal vector plots of TTRM thermal demagnetization (in specimen coordinates); Right: comparison of unblocking temperature spectra of NRM and TRM decay. Grey rectangles: temperatures used for TTRM paleointensity calculation.

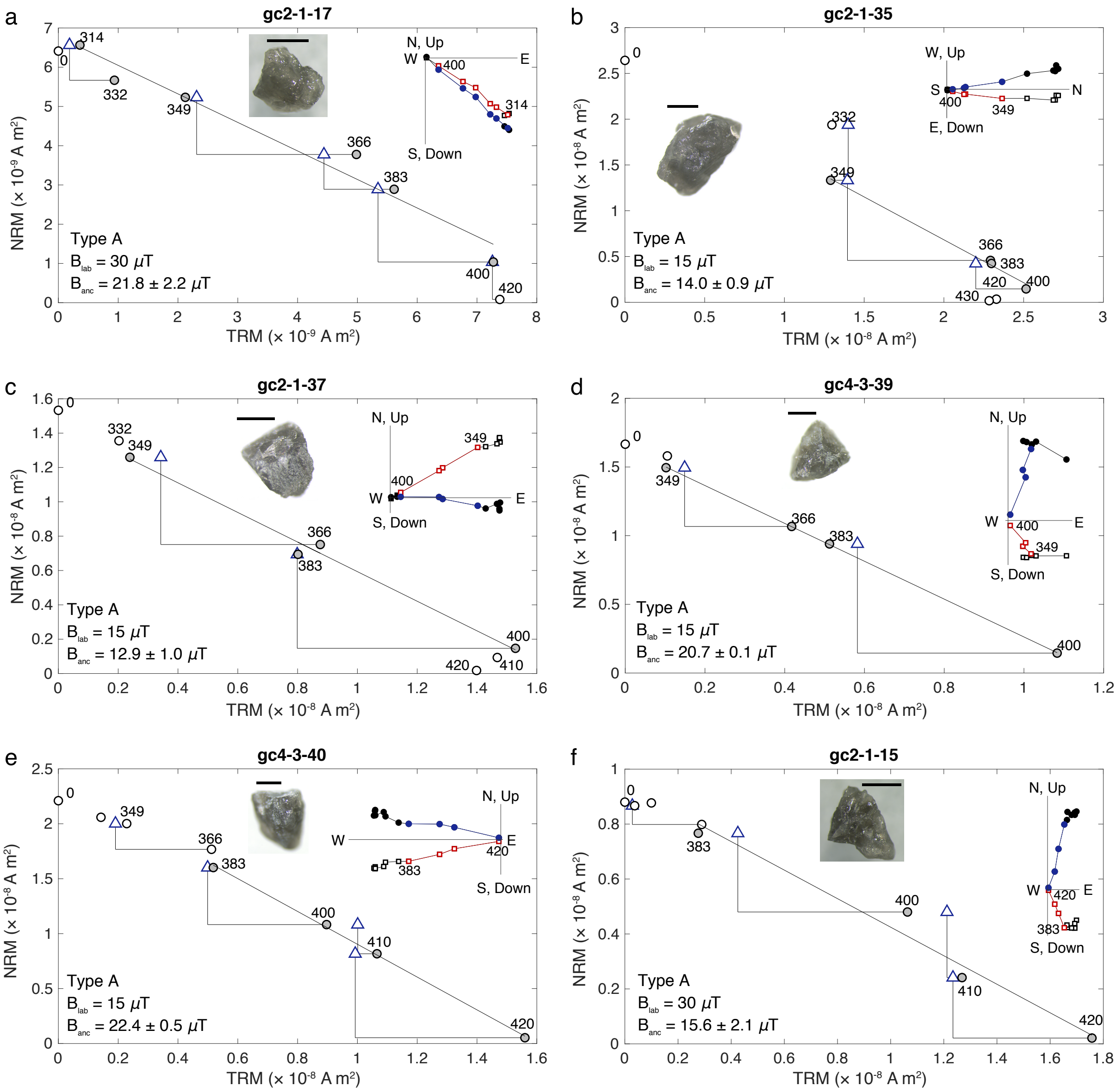

Supplementary Figure 7. Additional Thellier-Coe paleointensity measurement on single plagioclase crystals from the GMLC anorthosites. See caption of Figure 2 in the main text for explanation of symbols. The quality assessment of the result is labeled in the lower left for each sample.

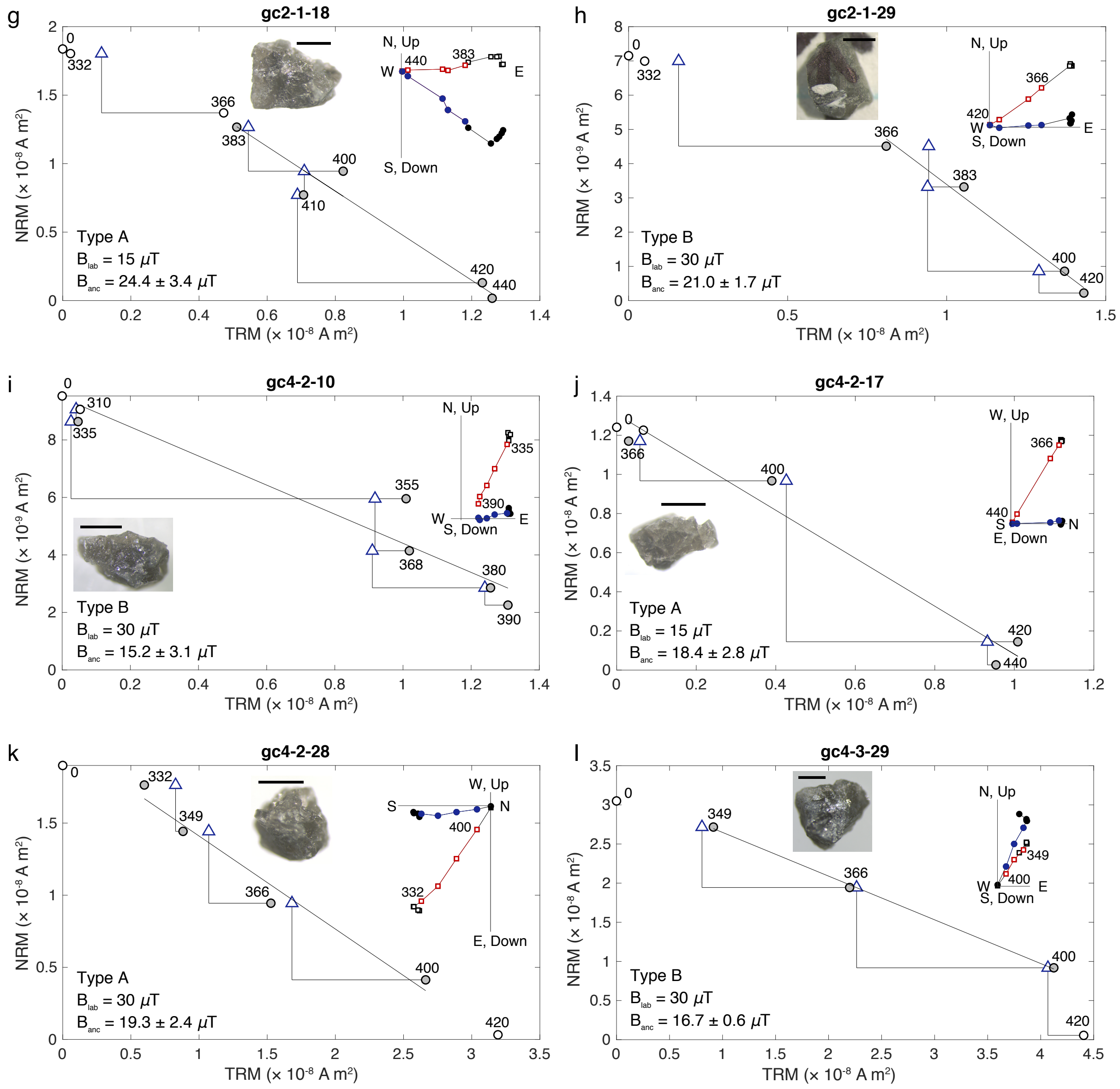

Supplementary Figure 7 continued.

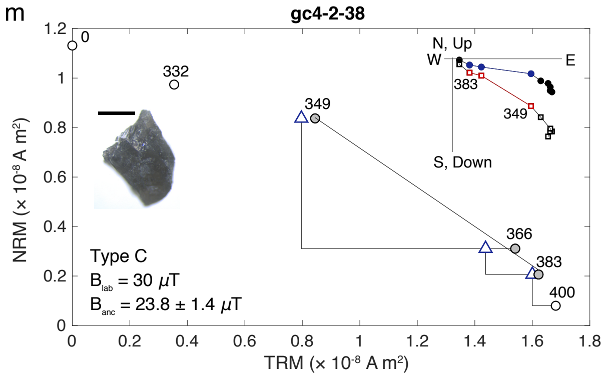

Supplementary Figure 7 continued.

4.0 Ediacaran to Cryogenian paleointensity data base.

Here we summarize Ediacaran to Cryogenian paleointensity data that have become available since publication of the Bono et al.[5] Precambrian paleointensity database. These are mainly from relatively rapidly cooled rocks (dikes, sills, and basalt flows), and in one case sedimentary basaltic clasts from such rocks. All studies are of bulk rocks, and the occurrence of MD grains leaves open the possibility of recording bias[6–8]. Thellier double heating techniques remain the gold standard for paleointensity studies[6–8]. Successful Thellier experiments have been reported, but because of alteration in nature and in the laboratory, and the presence of nonideal magnetic carriers that are ubiquitous in most whole rock samples, such data are few in number. For example only a single cooling unit result (with n=2) is available for 720 Ma, and data measured on only 2 samples from trachyte-basalt conglomerate clasts with possible ages of 550 Ma are available to bound the time interval considered here. Accordingly, some authors also report results from microwave, Shaw, and/or Wilson techniques to support the scarce Thellier results. These techniques sometimes yield results similar to Thellier data[9]. But, they are generally viewed as of lower reliability because of a series of unknowns, including those relating alternating magnetic fields or microwave energies to the fundmental thermal processes that govern the acquisition of TRMs. Available experiments highlight that these uncertainties are related to magnetic grain size/domain state distributions in whole rocks[10], which are usually inadequately defined. These unknowns could explain why there are sometimes large differences between values derived from these techniques relative to Thellier results[11]. As described below, we recalculated the mean paleointensity for each method according to cooling unit (dike, sill, and flow). We require results to be replicated within a cooling unit (i.e., $n \geq 2$). The determination of age follows the original authors' description, except where we note that in the absence of direct dating, the age of clasts in conglomerates is, *sensu stricto*, only the youngest possible age (i.e., the sedimentary age of the conglomerate itself).

Franklin Dikes (∼720 Ma). The Franklin mafic dikes are exposed in the Canadian Arctic and Greenland. Samples studied by Lloyd et al.[12] are expected to represent near instantaneous records of the field. Paleointensity measurements were conducted on bulk samples. SEM analyses indicate the presence of large MD low titanium magnetite 10's to 100's of μm in size, whereas mean magnetic hysteresis parameters are suggestive of MD to PSD domain states. Acceptable Thellier results with $n \geq 2$ are available from only a single site (BP). Replicated results are also reported using microwave and Shaw paleointensity results (3 and 6 dikes, respectively) are too few in number to average secular variation.

Skinner Cove volcanics (∼550 Ma). The volcanics of the Skinner Cove of Newfoundland are a series of mafic flows and trachyte flows and an intraformational conglomerate with igneous clasts. Thallner at al.[13] report that the magnetic carriers are titanomagnetite with evidence for varying degrees of oxidation (titanomaghemite formation). Acceptable Thellier results are available from only two clasts in the intraformational conglomerate. These are apparently selected from other

clasts which are overprinted from the same conglomerate[14]. Uncertainties associated with these data are that they have not been directly dated and that they appear to have considerable MD components (Figure 2e of Thallner et al.[13]). Results from 4 cooling units using microwave techniques are available, as well as 8 clasts from the conglomerate; here we average the latter into one unit to obtain 5 independent field recordings. These include results from one flow (SC01) that has SD to PSD average magnetic hysteresis properties. Similarly, results are available from 5 units using the Shaw technique, and include site SC01. Irrespective of reliability concerns, these results are too few in number to record the time-averaged geomagnetic field. Specifically, the uncertainty regions extend from ultralow field values to higher fields, and therefore these data alone are insufficient to detect a rise in field strength following the Ediacaran ultralow field[5]. Nevertheless, the mean values provide some hints to field history consistent with the PDMs of late Ediacaran/earliest Cambrian age (Figure 3).

Grenville dikes (∼584-598 Ma). Ediacaran age basaltic dikes with varying degrees of preservation are exposed in southweastern Ontario. Magnetic carriers are titanomagnetite, and SEM photos document the presence of MD grains, but also exsolution of magnetite in some dikes that could be in the PSD or SD state[15]. Some of these dikes were studied by Thallner et al.[15], but no acceptable Thellier results were recovered. Four dikes yielded acceptable microwave results and three Shaw data (we do not consider pseudo-Thellier results). While no Thellier results are available, these few instantaneous readings are consistent with a prolonged ultralow Ediacaran field.

Ukrainian traps and baked tuffs (561-580 Ma). Thick Ediacaran basaltic lavas (traps) and tuffs are found in Ukraine and their paleointensity records were reported by Shcherbakova et al.[15]. Magnetic minerals include mainly magnetite produced from high temperature oxidation, but also large (hundreds of μm) titanomagnetite grains and hematite, with magnetic hysteresis properties ranging from PSD to MD. Robust Thellier results are available from 4 flows. Thellier results are also available from baked tuffs adjacent to one flow and these are combined here into one time unit. Microwave and Wilson technique results are also available from most of these sites. While these data are too few in number to record the time-averaged field, all four estimates suggest a very weak field, consistent with an Ediacaran ultralow field[5].

5.0 References

1. Roggenthen, W. M., Fischer, J. F., Napoleone, G. & Fischer. A. G. Paleomagnetism and age of mafic plutons, Wichita Mountains, Oklahoma. *Geophys. Res. Lett.* **8**, 133-136, (1981).
2. Hanson, R. E. Puckett Jr, R. E., Keller, G. R., Brueseke, M. E. Bulen, C. L., Mertzman, S. A., Finegan, S. A. & McCleery, D. A. Intraplate magmatism related to opening of the southern Iapetus Ocean: Cambrian Wichita Igneous Province in the southern Oklahoma Rift Zone. *Lithos* **174**, 57-70 (2013).
3. Dunlop, D. J. Theory and application of the Day plot (Mrs/Ms versus Hcr/Hc) 1. Theoretical

curves and tests using titanomagnetite data. *J. Geophys. Res.* **107**, EPM 4-1 - EPM 4-22 (2002).

4. Watson, G. A. Large sample theory of the Langevin distributions. *J. Stat. Plann. Inference* **8**, 245-256 (1983).
5. Bono, R. K., Tarduno, J. A., Nimmo, F., & Cottrell, R. D. Young inner core inferred from Ediacaran ultra-low geomagnetic field intensity. *Nat. Geosci.* **12**, 143-147 (2019).
6. Tarduno, J. A., & Smirnov A. V. The paradox of low field values and the long-term history of the geodynamo, in Timescales of the Paleomagnetic Field, edited by J. E. T. Channell et al., Geophys. Monogr. Ser., vol. 145, pp. 75–84, AGU, Washington, D. C. (2004).
7. Smirnov, A. V., & Tarduno J. A.Thermochemical remanent magnetization in Precambrian rocks: Are we sure the geomagnetic field was weak? *J. Geophys. Res.* **110**, B06103 (2005).
8. Smirnov, A. V., Kulakov, E. V., Foucher, M. S. & Bristol, K. E. Intrinsic palaeointensity bias and the long-term history of the geodynamo. *Sci. Adv.* **3**, e1602306 (2017).
9. Suttie, N., Holme, R., Hill, M. J., Shaw, J., Consistent treatment of errors in archaeointensity implies rapid decay of the dipole prior to 1840. *Earth Planet. Sci. Lett.* **304** 13-21 (2011).
10. Lerner, G. A., Smirnov, A V., Surovitckii, L. V. & Piispa, E. J., Nonheating methods for absolute paleointensity determination: Comparison and calibration using synthetic and natural magnetite-bearing samples. *J. Geophys. Res.* **122**, 1614-1633 (2017).
11. Cottrell, R. D., Tarduno, J. A. & Roberts, J. The Kiaman Reversed Polarity Superchron at Kiama: Toward a field strength estimate based on single silicate crystals. *Phys. Earth Planet. Inter.* **169**, 49-58 (2008).
12. Lloyd, S. J., Biggin, A. J., Halls, H., & Hill, M. J. First palaeointensity data from the Cryogenian and their potential implications for inner core nucleation age. *Geophys. J. Int.* **226**, 66-77, (2021).
13. Thallner, D., Biggin, A. J., McCausland, P. J. & Fu, R. R. New paleointensities from the Skinner Cove Formation, Newfoundland, suggest a changing state of the geomagnetic field at the Ediacaran-Cambrian transition. *J. Geophys. Res.* **126**, e2021JB022292 (2021).
14. McCausland, P. J. A., & Hodych, J. P., Paleomagnetism of the 550 Ma Skinner Cove volcanics of western Newfoundland and the opening of the Iapetus Ocean. *Earth Planet. Sci. Lett.* **163**, 15-29 (1998).
15. Thallner, D., Biggin, A. J., & Halls, H.C. An extended period of extremely weak geomagnetic field suggested by palaeointensities from the Ediacaran Grenville Dykes (SE Canada). *Earth Planet. Sci. Lett.* **568**, 117025 (2021).
16. Shcherbakova, V., Bakhmutov, V., Thallner, D., Shcherbakov, V., Zhidkov, G., & Biggin, A. Ultra-low palaeointensities from East European Craton, Ukraine support a globally anomalous palaeomagnetic field in the Ediacaran. *Geophys. J. Int.* **220**, 1928-1946 (2020).
17. Veitch, R. J., Hedley, I. G. & Wagner, J.-J. An investigation of the intensity of the geomagnetic field during Roman times using magnetically anisotropic bricks and tiles. *Arch. Sci.* **37**, 359–373 (1984).

Sample	AF Range (mT)		N	MAD	D (º)	I (º)
gc2-3-1a	70	100	4	0.5	53.7	-17.4
gc2-3-1b	60	100	5	1	53.3	-17.6
gc2-3-3a	70	170	10	2	44.8	-13.6
gc2-3-4a	80	190	8	1.3	44.6	-13.8
gc2-3-5a	80	180	6	1.1	49	-13.7
gc2-3-6a	80	170	8	1.5	53.8	-13.4
gc4-3-1a	40	140	9	1.2	246.6	-0.9
gc4-3-2a	40	160	12	1.8	239	1
gc4-3-3a	50	140	10	2.1	239.6	1.5
gc4-3-4a	30	140	11	1.7	237.8	1.3
gc4-3-5a	30	140	11	1.7	243	1.5
gc4-3-6a	50	155	10	3.2	247.8	-7.4

Supplemental Table 1. AF demagnetization results of GMLC anorthosites whole rock specimens. AF Range: the range of alternating fields involved in the principal component ChRM fit. N: number of alternating field steps used in the ChRM fit. MAD: maximum angular deviation of the ChRM fit. D: ChRM declination; I: ChRM inclination. All fits are anchored to the origin of the orthogonal vector plot.

Sample	Highest T_{ub} (℃)	Acceptance	Temp Range (℃)		N	B (μT)	σ_B (μT)	Comment
gc2-1-3	500	accept	400	450	3	12.9	9.5	-
gc2-1-9	415	reject	-	-	-	-	-	not enough points
gc2-2-3	415	reject	-	-	-	-	-	offset from origin; not enough points
gc2-2-4	475	reject	-	-	-	-	-	offset from origin
gc2-3-2	400	reject	-	-	-	-	-	offset from origin
gc4-1-1	435	accept	355	400	3	7.3	0.7	-
gc4-1-7	510	reject	-	-	-	-	-	offset from origin
gc4-2-1	450	reject	-	-	-	-	-	altered
gc4-3-7	520	accept	400	450	3	21.4	8.3	a little offset from origin

Supplemental Table 2. TTRM results of plagioclase crystals from the GMLC anorthosites. Highest T_{ub}: the highest unblocking temperature of the sample. Temp Range: the range of unblocking temperature involved in the paleointensity estimation. B: the estimated paleointensity. σ_B: the standard deviation of the estimated paleointensity. TTRM results are rejected because of the following reasons: the orthogonal plot for the NRM or TRM demagnetization doesn't trend to the origin; the magnetization drops too quickly to give enough points to yield a reliable estimate; and/or the sample altered during the experiment.

Sample	Temp Range			N	MAD	δ_{pca}	Slope	$Slope_{err}$	R^2	B_{lab} (μT)	B_{anc} (μT)	B_{err} (μT)	DANG	FRAC	Beta	f	g	q	Ani Temp (°C)	Ani Factor	Type	Explanation for B and C type
gc2-1-15	383	420	+O	4	3.1	1.7	-0.519	0.069	0.965	30	15.6	2.1	9.1	0.66	0.134	0.80	0.639	3.84	400	1.3	A	
gc2-1-17	314	400	+O	6	2.3	1.1	-0.727	0.072	0.961	30	21.8	2.2	6.0	0.78	0.099	0.78	0.757	5.94			A	
gc2-1-18	383	440	+O	5	3.7	0.6	-1.627	0.229	0.942	15	24.4	3.4	7.9	0.57	0.140	0.59	0.516	2.16			A	
gc2-1-29	366	420	+O	4	2.5	1.6	-0.700	0.058	0.986	30	21.0	1.7	17.7	0.52	0.082	0.42	0.579	2.92			B	DRAT = 17.7
gc2-1-35	349	400	+O	4	2.7	3.4	-0.936	0.057	0.993	15	14.0	0.9	6.2	0.41	0.061	0.46	0.353	2.66			A	
gc2-1-36	349	400	+O	4	3.2	0.9	-0.866	0.025	0.998	15	13.0	0.4	7.2	0.61	0.028	0.81	0.604	17.32			A	
gc2-1-37	349	400	+O	4	3.2	2.2	-0.862	0.068	0.988	15	12.9	1.0	6.1	0.63	0.079	0.76	0.495	4.80			A	
gc4-1-39	420	474	+O	6	3.8	5.0	-1.062	0.068	0.983	15	15.9	1.0	5.6	0.41	0.064	0.38	0.742	4.42	420	1.4	A	
gc4-1-47	349	420	+O	4	3.2	1.7	-0.405	0.021	0.995	30	12.2	0.6	3.7	0.70	0.052	0.91	0.628	10.93	400	1.0	A	
gc4-2-10	335	390	+O	5	5.5	5.6	-0.506	0.103	0.879	30	15.2	3.1	7.7	0.60	0.204	0.67	0.586	1.94			B	R2 = 0.879
gc4-2-17	366	440	+O	4	0.7	0.4	-1.226	0.189	0.953	15	18.4	2.8	5.2	0.88	0.154	0.87	0.437	2.47	420	1.1	A	
gc4-2-28	332	400	+O	4	2.9	1.7	-0.645	0.080	0.969	30	19.3	2.4	9.3	0.66	0.124	0.65	0.627	3.29			A	
gc4-2-37	349	420	+O	6	2.6	1.4	-1.107	0.038	0.995	30	33.2	1.1	3.2	0.81	0.034	0.92	0.749	19.93	400	0.7	A	
gc4-2-38	349	383	+O	3	3.1	2.7	-0.792	0.048	0.996	30	23.8	1.4	10.3	0.49	0.060	0.41	0.234	1.61	366	1.2	C	N = 3, DRAT = 10.3
gc4-3-29	349	400	+O	3	1.6	0.3	-0.558	0.019	0.999	30	16.7	0.6	2.9	0.48	0.034	0.56	0.486	8.10			B	N = 3
gc4-3-39	349	400	+O	4	3.3	1.7	-1.378	0.008	1.000	15	20.7	0.1	4.2	0.52	0.006	0.82	0.546	81.06	383	0.8	A	
gc4-3-40	383	420	+O	4	3.5	2.1	-1.493	0.036	0.999	15	22.4	0.5	5.6	0.64	0.024	0.65	0.616	16.74	400	0.8	A	

Supplementary Table 3. Thellier-Coe paleointensity results from GMLC single plagioclase crystals. Parameters follow Bono et al.[5] and references therein. Sample; Temp Range: the unblocking temperatures used in the paleointensity fit (+O indicates origin is included in principal component (PCA) fit); N, the number of temperature steps used to determine the line fit of the Arai plot; MAD, maximum angular deviation of the PCA fit; δ_{pca}, the angle between the PCA component for the selected temperature range including and not including the origin; Slope and $Slope_{err}$, the slope and 1σ uncertainty of the line fit; R^2, paleointensity regression coefficient; B_{lab}, the laboratory applied field; B_{anc} and B_{err}, the paleointensity and 1σ uncertainty; DRAT, the maximum absolute difference produced by a pTRM check normalized by the length of the best fit line; FRAC, the fraction of NRM statistic; Beta, relative standard deviation of the best fit line; f, the fraction of NRM used in the line fit; g, gap factor; q, quality factor; Ani Temp, the temperature step for anisotropy test; Ani Factor, anisotropy factor (Veitch et al.)[17]. Type: classification of the paleointensity data into groups based on reliability criteria met; Explanation for samples in the B and C categories.

Virtual Dipole Moment

VDM (× 10^{22} A m^2)	σ_{VDM} (× 10^{22} A m^2)	Age (Ma)	Number of Cooling Unit	Method	Reference
0.9	0.9	720	1	Thellier	Lloyd et al.[12]
0.97	0.34	561	1	Thellier	Shcherbakova et al.[16]
1.03	0.19	561	1	Thellier	Shcherbakova et al.[16]
0.95	0.27	561	1	Thellier	Shcherbakova et al.[16]
0.52	0.23	580	1	Thellier	Shcherbakova et al.[16]
1.5	0.1	550	1	Thellier	Thallner et al.[13]
1.0	0.2	720	3	Microwave	Lloyd et al.[12]
0.6	0.1	584	1	Microwave	Thallner et al.[15]
0.4	0.2	598	1	Microwave	Thallner et al.[15]
0.7	0.2	587	1	Microwave	Thallner et al.[15]
0.8	0.2	585	1	Microwave	Thallner et al.[15]
1.05	0.27	561	1	Microwave	Shcherbakova et al.[16]
1.1	0.05	561	1	Microwave	Shcherbakova et al.[16]
2.0	0.9	550	5	Microwave	Thallner et al.[13]
1.2	0.8	720	6	Shaw	Lloyd et al.[12]
0.8	0.3	587	1	Shaw	Thallner et al.[15]
0.8	0.1	585	1	Shaw	Thallner et al.[15]
1.1	0.8	550	5	Shaw	Thallner et al.[13]
0.9	0.28	561	1	Wilson	Shcherbakova et al.[16]
0.95	0.1	561	1	Wilson	Shcherbakova et al.[16]
1.3	0.22	561	1	Wilson	Shcherbakova et al.[16]
0.57	0.18	580	1	Wilson	Shcherbakova et al.[16]

Paleomagnetic Dipole Moment

PDM (× 10^{22} A m^2)	σ_{PDM} (× 10^{22} A m^2)	Age (Ma)	Number of Determinations	Method	Ref
0.67	0.27	565	17	Thellier	Bono et al.[5]
3.5	0.9	532	17	Thellier	This study

Supplemental Table 4. Cryogenian-earliest Cambrian paleointensity data. VDM/PDM and $\sigma_{VDM}/\sigma_{PDM}$, Virtual/Paleomagnetic Dipole moment and 1σ uncertainty; Age; Number of cooling units or determinations used in dipole moment estimation; Method, paleointensity method used in the study.
